# Treatment-related hearing loss in weekly versus triweekly cisplatin chemoradiation for head and neck cancer

**DOI:** 10.1007/s00405-024-08880-x

**Published:** 2024-09-06

**Authors:** A. V. M. Burger, C. W. Duinkerken, K. E. van Sluis, J. P. de Boer, A. Navran, C. P. Lanting, K. Jóźwiak, W. A. Dreschler, A. J. M. Balm, C. L. Zuur

**Affiliations:** 1 Department of Head and Neck Surgery and Oncology, The Netherlands Cancer Institute Antoni van Leeuwenhoek, Plesmanlaan 121, Amsterdam, 1066 CX The Netherlands; 2 Department of Otolaryngology and Head and Neck Surgery, Leiden University Medical Centre, Albinusdreef 2, Leiden, 2333 ZA The Netherlands; 3 Department of Medical Oncology, The Netherlands Cancer Institute, Amsterdam, The Netherlands; 4 Department of Radiation Oncology, The Netherlands Cancer Institute, Amsterdam, The Netherlands; 5 Department of Otorhinolaryngology, Donders Institute for Brain, Cognition and Behaviour, Radboud University Medical Centre, Nijmegen, The Netherlands; 6 Institute of Biostatistics and Registry Research, Brandenburg Medical School Theodor Fontane, Neuruppin, Germany; 7 Department of Audiology, Amsterdam University Medical Center, Amsterdam, the Netherlands; 8 Department of Maxillofacial Surgery, Amsterdam University Medical Center, Amsterdam, The Netherlands

**Keywords:** Chemoradiotherapy, Cisplatin, Ototoxicity, Hearing loss, Head and neck

## Abstract

**Purpose:**

Cisplatin-induced hearing loss is a common side effect in patients treated with cisplatin-based chemoradiation (CRT) for head and neck squamous cell carcinoma. The extent of hearing loss after concurrent CRT was compared between triweekly (3 × 100 mg/m^2^) and weekly (7 × 40 mg/m^2^) cisplatin CRT.

**Method:**

This retrospective cohort study was conducted in the Antoni van Leeuwenhoek Hospital and included 129 patients with cisplatin-based CRT for head and neck cancer (72 treated in the triweekly and 57 in the weekly regimen). Baseline and follow-up pure tone audiometry was conducted to assess hearing loss. Clinically relevant hearing loss was defined as a decline upon treatment of ≥ 10 decibel at a pure tone average 1-2-4 kHz and/or 8-10-12.5 kHz.

**Results:**

The incidence of clinically relevant cisplatin CRT induced hearing loss was 42% in the triweekly versus 19% in the weekly group (*p* < 0.01). The mean threshold shift at a pure tone average (PTA) 1-2-4 kHz was 9.0 decibel in the triweekly compared to 4.3 decibel in the weekly CRT group (*p* < 0.01). At PTA 8-10-12.5 kHz, the incidence of clinically relevant hearing loss was 75% in the triweekly compared to 74% in the weekly CRT group (*p* = 0.87). The mean threshold shift at PTA 8-10-12.5 kHz was 20.2 decibel versus 15.6 decibel, respectively (*p* = 0.07).

**Conclusion:**

Cisplatin-dose reduction to a weekly cisplatin CRT regimen for head and neck cancer may reduce the incidence of clinically relevant hearing loss at frequencies vital for speech perception.

## Introduction

Cisplatin is a widely used anti-cancer drug in treating numerous types of cancers, including head and neck squamous cell carcinoma (HNSCC). Advanced HNSCC is often treated with (adjuvant) high-dose cisplatin chemoradiation (CRT), i.e., 3 × 100 mg/m^2^, as adding cisplatin to RT leads to improved survival rates in these patients compared to radiotherapy alone [1–3]. However, high-dose cisplatin may cause considerable side effects, including acute toxicities such as nausea, stomatitis, myelosuppression [1, 2], nephrotoxicity, neurotoxicity, i.e., peripheral nerve toxicity, and hearing loss [2, 4].

Hearing loss may occur when cisplatin damages various cochlear structures, including the outer and inner hair cells, stria vascularis, and spiral ganglion cells. Several biological processes are involved in developing cisplatin-induced hearing loss (CIHL), amongst others, releasing toxic reactive oxygen species and depleting the cochlea’s protective antioxidants [4–8]. Furthermore, the development of CIHL is influenced by several co-occurring risk factors, including a cochlear radiation dose of more than 30 Gy (Gy) [9, 10], and favorable pre-treatment hearing capacity, as often seen in younger patients [8, 11–14].

The clinical presentation of CIHL is characterized by symmetric and irreversible sensorineural hearing loss (SNHL) starting at extended high-frequencies and progressing to lower frequencies with continued treatment [4, 5, 15]. However, due to the heterogeneity in treatment schedules and used definitions of ototoxicity in studies conducted so far, it is hard to report the incidence of CIHL precisely [4, 10, 12, 16].

It is widely accepted that a cumulative concurrent cisplatin dose of ≥ 200 mg/m^2^ is a prerequisite for its anticancer efficacy in advanced HNSCC patients [17, 18]. However, approximately 30% of the patients suffer from cisplatin-related dose-limiting toxicities [19–21]. Therefore, an alternative CRT schedule for HNSCC has been designed to reduce toxicity and increase compliance to this intensive treatment regimen. The standard of care triweekly CRT schedule (100 mg/m^2^ cisplatin, days 1, 22, and 43; further referred to as “triweekly CRT schedule”) was adapted to a weekly CRT schedule (40 mg/m^2^ cisplatin, weekly during seven consecutive weeks; further referred to as “weekly CRT schedule”). Earlier research showed that the weekly schedule gives less toxicities such as nephrotoxicity and neutropenia [2, 22], however these studies did not elaborate on the difference in hearing loss between both schedules. Therefore, the aim of our study was to compare hearing loss in HNSCC patients treated with weekly and triweekly high-dose cisplatin CRT.

## Methods

### Study design and subjects

This is a retrospective cohort study with HNSCC patients treated with radiotherapy (five times a week for seven weeks, with a cumulative radiotherapy dose of 70 Gy) and concomitant intravenous cisplatin in a cumulative dose of at least 200 mg/m^2^. All patients were treated at the Netherlands Cancer Institute. The triweekly CRT group received 100 mg/m^2^ cisplatin every three weeks (on days 1, 22 and 43). Most of these patients were treated between 1999 and 2004 [23] and 2018 and 2020. The weekly CRT group received a weekly cisplatin dose of 40 mg/m^2^ (on days 1, 8, 15, 22, 29, 36 and 43) between 2020 and 2023. Due to dose limiting toxicities, some patients did not complete the full planned cisplatin schedule and continued treatment with RT only. We included patients that received a cumulative dose of 200 mg/m^2^ or more, as this is the minimum cumulative dose of cisplatin needed for increased CRT-related anticancer efficacy. In view of future informed consent for patients we wished to assess treatment-related hearing loss in patients receiving ≥ 200 mg/m^2^ cisplatin CRT.

Only patients with both baseline and follow-up audiometry were included in the current study. Patients treated with a radiation dose of ≥ 30 Gy at the cochlea were excluded from the analysis, as a RT dose of ≥ 30 Gy to the inner may also cause sensorineural hearing loss [24, 25]. This study was approved by the Institutional Review Board of the Netherlands Cancer Institute (ID IRBd22-261) and executed according to the Declaration of Helsinki.

### Audiometric analysis

Pure tone audiometry (0.125–8.0 kHz hearing level (HL)) and extended high-frequency audiometry (8.0–12.5 kHz sound pressure level (SPL)) were performed at baseline and at a median of 6 weeks [Q1-Q3: 5–7 weeks] after treatment. Air conduction (AC) and bone conduction (BC) thresholds (0.5–4 kHz HL) were measured in a sound-proof booth using the Decos Audiology Workstation. If BC thresholds were ≥ 10 dB better than AC thresholds on 0.5, 1, 2, or 4 kHz, BC thresholds were used. In case the AC threshold in extended high-frequency was missing on 8.0 kHz SPL, thresholds were imputed by taking the threshold at 8 kHz HL from pure tone audiometry and adding 13 dB, taking the reference equivalent threshold sound pressure level for 8 kHz as defined in ISO 389-1 [26]. If a patient’s hearing threshold was beyond the audiometer’s maximum output at the follow-up measurement and therefore not testable, this threshold was computed by adding 10 dB to the maximum measurable threshold (i.e., 110 + 10 = 120 dB, depending on the settings of the audiometer).

Two Pure Tone Averages (PTA’s) were calculated. The first PTA is the mean threshold (dB HL) of the frequencies 1, 2, and 4 kHz HL (PTA 1-2-4 kHz), as these frequencies are closely related to speech perception. The other PTA at frequencies 8, 10, and 12.5 kHz SPL (PTA 8-10-12.5 kHz) represents the perception of high sounds as in music but also enhances speech perception in noise [27, 28]. PTA threshold shifts were calculated as post-treatment hearing threshold minus pre-treatment hearing threshold. Clinically relevant CIHL was defined as a threshold shift of ≥ 10 dB at these PTA’s in at least one ear.

Furthermore, CIHL was graded with three grading scales: the International American Speech-Language-Hearing Association (ASHA) grading scale for hearing loss due to ototoxic drugs [29], the Common Terminology Criteria for Adverse Events (CTCAE) version 5.0 (based on the threshold shifts up to 8 kHz HL) [30], and the TUNE criteria [31] (Appendix A).

### Statistical analysis

Baseline characteristics between the two cisplatin groups were compared using Chi-square, Fisher’s exact, and Mann-Whitney-U tests. Incidence of clinically relevant hearing loss and indication for hearing aids de novo were compared between the two cisplatin groups using a Chi-square test. The scores on the grading scale for ototoxicity were compared between the two groups using a linear-by-linear test. The threshold shifts in hearing after therapy (i.e., the difference between post- and pre-treatment hearing threshold) at PTA1-2–4 kHz HL and PTA 8-10-12.5 kHz SPL were compared between the two cisplatin groups using a random intercept linear mixed model. The outcome was expressed per ear, as audiometry data was available for both ears, and nested at patient level. Univariable models were run separately for all PTAs with cisplatin schedule and each of the following covariates: baseline age, sex, cumulative cisplatin dose/m^2^, cochlear radiation dose, and baseline hearing level at PTA 1-2-4 kHz. If the *p*-value for a covariate was < 0.1, this covariate was added to the multivariable model with cisplatin schedule. Additionally, interaction terms between significant values in the univariable analysis were added to multivariable models and retained if significant. Data analysis was performed using IBM SPSS Statistics 27. A *p*-value of < 0.05 was considered statistically significant.

## Results

### Subjects

One hundred ten patients were treated in the triweekly CRT cohort. Thirty-eight patients were excluded because of baseline audiometry missing (*n* = 5), follow-up audiometry missing (*n* = 16), dose-limiting toxicity leading to a cumulative cisplatin dose < 200 mg/m^2^ (*n* = 2), and cochlear radiation dose ≥ 30 Gy (*n* = 15), resulting in 72 evaluable patients. Sixty-nine patients were treated with the weekly CRT schedule. Twelve of them were excluded from the analysis because of baseline audiometry missing (*n* = 3), follow-up audiometry missing (*n* = 1), dose-limiting toxicity leading to a cumulative cisplatin dose < 200 mg/m^2^ (*n* = 5), and radiotherapy dose on cochlea ≥ 30 Gy (*n* = 3), resulting in 57 evaluable patients.

The baseline characteristics of both treatment groups are shown in Table 1. Patients in the triweekly CRT group were relatively younger (56.2 versus 60.7 years old, *p* < 0.01). In the weekly CRT group, 65% of all patients were treated for oropharyngeal cancer compared to 36% in the triweekly CRT group. The mean cochlear radiotherapy dose was higher in the triweekly CRT group, namely 14.2 Gy versus 8.1 Gy (*p* < 0.01).

**Table 1:** Baseline characteristics. (a) Chi-Square test; (b) independent samples T-test; (c) Fisher’s exact test, a p-value of < 0.05 is considered statistically significant. Abbreviation: CRT: chemoradiation

		Triweekly CRT group (*n* = 72)	Weekly CRT group (*n* = 57)	*p*-value
Sex (%)	Male	56 (78)	40 (71)	0.33^a^
	Female	16 (22)	17 (29)	
Age (years) (mean, SD)		56.2 (± 9.9)	60.9 (± 8.4)	< 0.01^b^
Tumor localization (%)	Oropharyngeal	26 (36)	37 (65)	< 0.01^c^
	Oral cavity	9 (13)	8 (14)	
	Laryngeal	21 (29)	3 (5)	
	Hypopharyngeal	13 (18)	5 (9)	
	Nasopharyngeal	2 (3)	3 (5)	
Unknown primary		0 (0)	1 (2)	
	Other	1 (1)	0 (0)	
Cumulative cisplatin dose (%)	300 mg/m^2^	67 (93)	0 (0)	n.a.
	280 mg/m^2^	0 (0)	38 (67)	
	240 mg/m^2^	0 (0)	13 (23)	
	200 mg/m^2^	5 (7)	6 (10)	
Cochlear radiation dose (gray) (mean, SD)		14.2 (7.8)	8.1 (7.6)	< 0.01^b^

### Audiometry results

**Fig. 1:**
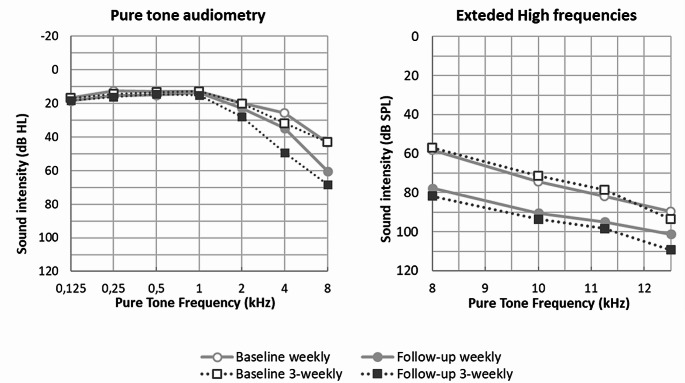
Baseline and follow-up average audiometry for both treatment groups. (**A**). Pure tone audiometry. (**B**). Extended high frequency audiometry. Abbreviations dB: decibel, HL: hearing level; SPL: sound pressure level; kHz: kiloHertz

The audiometric data of all patients in both cohorts is presented in Fig. 1; Table 2. The mean threshold shift at PTA 1-2-4 kHz was 9.0 (± 9.9) dB in the triweekly CRT group and 4.3 (± 8.2) dB in the weekly CRT group (*p* < 0.01). The mean threshold shift at PTA 8-10-12.5 kHz was 20.2 (± 16.4) dB in the triweekly CRT group and 15.6 (± 14.0) dB in the weekly CRT group (*p* = 0.07). At the frequencies of 1-2-4 kHz, we observed clinically relevant CIHL, defined as a threshold shift of 10 dB or more, in 31 out of 72 patients (42%) from the triweekly CRT group and 11 out of 57 patients (19%) from the weekly CRT group (*p* < 0.01). At frequencies of 8-10-12.5 kHz, clinically relevant CIHL was observed in 54 out of 72 patients (75%) from the triweekly CRT group and 42 out of 57 patients (74%) from the weekly CRT group (*p* = 0.87). Significantly higher grading scale scores were observed in the triweekly CRT schedule compared to the weekly CRT schedule on both the CTCAE and TUNE (both *p* < 0.01). However, hearing loss, as defined by the ASHA criteria, was not significantly different between both groups (*p* = 0.81). Furthermore, more patients in the triweekly CRT group had an indication for hearing aids de novo after treatment compared to the weekly CRT schedule (36% versus 14%, *p* < 0.01).

**Table 2:** Incidence of ≥ 10 dB hearing loss at PTA 1-2-4 kHz HL and PTA 8-10-12.5 kHz SPL, threshold shift at both PTAs, incidence of an indication for hearing aids de novo (PTA ≥ 35 dB after CRT and < 35 dB at baseline), and scores on grading scales for both CRT groups. (a) Chi-Square test; (b) Fisher’s exact test; (c) Linear mixed model; (d) Linear-by-linear association. A p-value of < 0.05 is considered statistically significant.Abbreviations: kHz: kiloHertz; SPL: sound pressure level; HL: hearing level; dB: decibel, ASHA: American Speech-Language-Hearing Association criteria; CRT: chemoradiation; CTCAE: common terminology criteria for adverse events

Variables		Triweekly CRT	Weekly CRT	*p*-value
		*n* = 72 patients	*n* = 57 patients	
Hearing loss at PTA 1-2-4 kHz HL				
	Incidence (%)	30 (42)	11 (19)	< 0.01^a^
	Hearing loss in dB per ear (mean, range)	9.0 (-10.0–50.0)	4.3 (-5.0–36.7)	< 0.01^c^
Hearing loss at PTA 8-10-12.5 kHz SPL				
	Incidence (%)	54 (75)	42 (74)	0.87^a^
	Hearing loss in dB per ear (mean, range)	20.2 (-15.2–60.0)	15.6 (-25.0–61.7)	0.06^c^
Indication for hearing aids (number of patients (%))		26 (36)	8 (14)	< 0.01^b^
Grading scales				
ASHA				0.81^d^
	no hearing loss (%)	9 (13)	10 (18)	
	grade A (%)	3 (4)	4 (7)	
	grade B (%)	58 (81)	36 (63)	
	grade C (%)	2 (3)	7 (12)	
CTCAE v5.0				< 0.01^d^
	grade 0 (%)	32 (44)	43 (75)	
	grade 1 (%)	6 (8)	3 (5)	
	grade 2 (%)	8 (11)	1 (2)	
	grade 3 (%)	26 (36)	10 (17)	
	grade 4 (%)	0 (0)	0 (0)	
TUNE				< 0.01^d^
	grade 0 (%)	9 (12)	9 (16)	
	grade 1a (%)	6 (8)	19 (33)	
	grade 1b (%)	3 (4)	0 (0)	
	grade 2a (%)	24 (33)	19 (33)	
	grade 2b (%)	4 (6)	1 (2)	
	grade 3 (%)	26 (36)	9 (16)	
	grade 4 (%)	0 (0)	0 (0)	

### Linear mixed model

Results of univariable and multivariable linear mixed model analyses are presented in Tables 3 and 4. The threshold shift in hearing after therapy at PTA 1-2-4 kHz HL was significantly higher by 3.5 dB in the triweekly CRT group compared to the weekly CRT group (estimate 3.55, 95% CI 0.15–6.95, *p* = 0.04) after adjustment for radiotherapy dose to the cochlea, age and baseline hearing level at PTA 1-2-4 kHz. The threshold shift was significantly higher with higher cochlear radiation dose (estimate 0.28, 95% CI 0.12–0.44, *p* < 0.01). However, the difference in threshold shift in hearing after therapy at PTA 8-10-12.5 kHz HL was smaller and not significant between the two cisplatin CRT schedules (estimate – 0.50, 95% CI -4.71–5.71, *p* = 0.85) after adjustment for baseline age, radiotherapy dose to the cochlea and baseline hearing level at PTA 1-2-4 kHz. The threshold shift at PTA 8-10-12,5 kHz was significantly smaller in older patients (estimate – -0.36., 95% CI -0.64 – -0.09, *p* = 0.01) and in patients with worse baseline hearing level at PTA 1-2-4 kHz (estimate – -0.21, 95% CI -0.39 – -0.05, *p* = 0.01). The threshold shift at PTA 8-10-12,5 kHz was significantly higher with higher cochlear radiation dose (estimate 0.33, 95% CI 0.08–0.58, *p* = 0.01). No significant interactions were found between the variables used in the multivariable model.

**Table 3:** Linear mixed model for PTA 1-2-4 kHz HL. A p-value < 0.05 is considered statistically significant. Abbreviations: CRT: chemoradiation; PTA: pure tone average

Variable		Univariable analysis			Multivariable analysis		
		Estimate	95% CI	*p*-value	Estimate	95% CI	*p*-value
Cisplatin schedule	Weekly CRT	Ref.					
	Triweekly CRT	4.75	1.78–7.71	< 0.01	3.55	0.15–6.95	0.04
Baseline age in years		-0.11	-0.27–0.05	0.17	0.00	-0.18–0.18	0.97
Sex	Female	Ref.					
	Male	2.14	-1.35–5.63	0.23			
Cumulative cisplatin dose/m^2^		0.02	-0.03–0.08	0.33			
Cochlear radiation dose in Gray		0.35	0.19–0.50	< 0.01	0.28	0.12–0.44	< 0.01
Baseline hearing level at PTA 1-2-4 kHz, dB HL		-0.10	-0.20–0.00		0.05	-0.21–0.01	

**Table 4:** Linear mixed model for PTA 8-10-12.5 kHz HL. P-value < 0.05 is considered statistically significant. Abbreviations: CRT: chemoradiation; PTA: pure tone average

Variable			Univariable analysis			Multivariable analysis		
			Estimate	95% CI	*p*-value	Estimate	95% CI	*p*-value
Cisplatin schedule	Weekly CRT		Ref.			Ref.		
	Triweekly CRT		4.67	-0.32–19.29	0.07	0.50	-4.71–5.71	0.85
Baseline age in years			-0.57	-0.82 – -0.32	< 0.01*	-0.36	-0.64 – -0.09	0.01
Sex		Female	Ref.					
		Male	-0.84	-6.60–4.93	0.77			
Cumulative cisplatin dose/m^2^			0.02	-0.06–0.10	0.47			
Cochlear radiation dose in Gray			0.40	0.15–0.65	< 0.01*	0.33	0.08–0.58	0.01
Baseline hearing level at PTA 1-2-4 kHz, dB HL			-0.35	-0.50 – -0.19	< 0.01*	-0.22	-0.39 – -0.05	0.01

## Discussion

Although the main goal of anticancer therapy remains to achieve better survival and loco-regional control, improving post-treatment quality of life by reducing treatment-related toxicity has become increasingly important [11, 32]. In HNSCC, weekly cisplatin CRT (7 cycles of 40 mg/m^2^ cisplatin during seven consecutive weeks) achieves similar survival rates when compared to triweekly CRT (3 cycles of 100 mg/m^2^ cisplatin every three weeks) [2, 22]. Also, it is accompanied by less cisplatin toxicities such as nephrotoxicity, neutropenia, and electrolyte disturbances [2, 22]. The objective of this study was to assess whether adopting a weekly CRT schedule reduces CIHL.

The implementation of weekly CRT may contribute to preserving hearing capacity and improving quality of life [11, 32]. In our research, the incidence of clinically relevant hearing loss of ≥ 10 dB at PTA 1-2-4 kHz, representing the perception of speech in noise, was found significantly higher in the triweekly CRT group compared to the weekly CRT group (42% versus 19%, *p* < 0.01), in agreement with previous studies [2, 33]. The 23%-point difference in the incidence, the marked difference in hearing-aid candidacy (36% versus 14%), CTCAE criteria (*p* < 0.01) and TUNE criteria (*p* < 0.01) indicate benefit of a weekly cisplatin regimen over a triweekly cisplatin regimen with respect to CRT-induced hearing loss in HNSCC patients. Consequently, a weekly cisplatin regimen might reduce adverse effects commonly observed in HNSCC patients’ health-related quality of life, including social isolation, anxiety, and depression [32, 34]. Careful interpretation of our data is warranted in view of the retrospective nature of our research. A limitation of this retrospective design was that the lack of speech audiometry in most patients, which would have provided valuable extra information about speech processing capacity prior and after CRT. However, a detailed description of data was, available for all patients, including audiometric hearing thresholds up to 12.5 kHz SPL, the cochlear radiation dose per ear, and the gradation of hearing loss as defined by different grading scales. Therefore, for monitoring CIHL, we recommend standard and extended high-frequency pure tone audiometry in all patients treated with high-dose CRT, at least before the start of treatment and approximately two to three months after the last CRT.

We found a significant association between cochlear radiation dose and CIHL, in agreement with previous studies that found a cochlear radiation dose ≥ 30 Gy to cause clinically relevant sensorineural hearing loss of ≥ 10 dB [9, 24]. Other literature advises to limit the radiation dose to the cochlea to ≤ 35 Gy [25, 35], however we chose to use the most strict cut-off value. The triweekly CRT group, mainly treated between 1999 and 2004, received a higher mean cochlear radiation dose, attributed to a difference in radiation techniques and planning in the years 1999–2004 when compared to more recently treated patients in both the weekly and triweekly CRT schedule (16.1 Gy versus 8.4 Gy). Despite the limitation of this time difference and difference in radiation technique, after correcting for the mean cochlear radiation dose in our multivariable analysis, significantly more hearing loss at PTA 1-2-4 was found in the triweekly compared to the weekly CRT group. Also, we found no significant difference in CIHL between patients in the triweekly groups treated between 1999 and 2004 and 2018–2020 on PTAs 1-2-4 (*p* = 0.08) and 8-10-12,5 (*p* = 0.36). Therefore we believe that it is justified to evaluate all triweekly patients as one cohort, regardless of the difference in treatment period.

Even though weekly CRT may decrease the incidence of cisplatin-CRT induced hearing loss, there is still a need for an otoprotectant in both treatment regimens. Recently, both systemic and topical (transtympanic) approaches have been studied to reduce CIHL with varying successes [11, 13, 36, 37]. Antioxidants are probably the most encouraging otoprotective agents, as they can neutralize the toxic formation of reactive oxygen species by cisplatin. Interestingly, the antioxidant sodium-thiosulphate (STS) can also inactivate cisplatin. When STS is injected into the middle ear (transtympanically), it may locally inactivate cisplatin without interfering with its systemic anticancer effect. In a recent phase I trial, this method was safe and feasible [38]. Its efficacy is currently studied further in a multicenter phase 3 randomized controlled setting (*CTIS 2023-503313-30-01)*. The current study shows that patients treated in both schedules are still prone to develop clinically relevant CIHL. Therefore, HNSCC patients treated in both the triweekly and the weekly CRT schedule are eligible to participate in our phase 3 trial regarding the efficacy of transtympanic STS against CIHL.

In conclusion, hearing capacity seems to be relatively preserved after treatment with a weekly cisplatin CRT regimen (7 cycles of 40 mg/m^2^ cisplatin during seven consecutive weeks) when compared to triweekly cisplatin CRT regimen (3 cycles of 100 mg/m^2^ cisplatin every three weeks) for HNSCC. However, both treatment schedules induce clinically relevant CIHL at extended high-frequencies, which impairs the quality of higher sounds (e.g., for music) and speech perception in noise [27, 28]. Currently a multicenter phase 3 study to evaluate the efficacy of transtympanic STS against CIHL is underway. Ultimately, these efforts should reduce CIHL and thereby increase the quality of life in HNSCC patients and survivors.

## Appendix A

**Table 5:** Grading scales that were used for the assessment of post-treatment platinum-related hearing loss. Abbreviations: ASHA: American Speech-Language-Hearing Association; CTCAE: common terminology criteria for adverse events

Grading scale	Definition of hearing loss
ASHA [29]	A) 20 dB decrease at any one tested frequency B) 10 dB decrease at any two adjacent test frequencies C) loss of response at three consecutive test frequencies where responses were previously obtained
CTCAE v5.0 (on a 1, 2, 4, 3, 6, and 8 kHz audiogram) [30]	Grade 1: Threshold shift of 15–25 dB averaged at 2 contiguous test frequencies in at least one ear OR Subjective change in hearing in the absence of documented hearing loss Grade 2: Threshold shift of > 25 dB averaged at 2 contiguous test frequencies in at least one ear Grade 3: Threshold shift of > 25 dB averaged at 3 contiguous test frequencies in at least one ear OR hearing aid or intervention indicated Grade 4: Decrease in hearing to profound bilateral loss (absolute threshold > 80 dB HL at 2 kHz and above); non-serviceable hearing
TUNE [31]	Grade 0: No hearing loss Grade 1a: Threshold shift ≥ 10 dB at [8-10-12.5] OR subjective changes in the absence of a threshold shift Grade 1b: Threshold shift ≥ 10 dB at [1–2–4] Grade 2a: Threshold shift ≥ 20 dB at [8-10-12.5] Grade 2b: Threshold shift ≥ 20 dB at [1-2-4] Grade 3: Hearing level ≥ 35 dB HL at [1-2-4] de novo Grade 4: Hearing level ≥ 70 dB HL at [1-2-4] de novo

## Data Availability

Research data are stored in an institutional repository and can be shared upon request to the corresponding author and after ethical clearance of the NKI Institutional Review Board.
